# The effect of missing data and imputation on the detection of bias in cognitive testing using differential item functioning methods

**DOI:** 10.1186/s12874-022-01572-2

**Published:** 2022-03-27

**Authors:** E. Nichols, J. A. Deal, B. K. Swenor, A. G. Abraham, N. M. Armstrong, K. Bandeen-Roche, M. C. Carlson, M. Griswold, F. R. Lin, T. H. Mosley, P. Y. Ramulu, N. S. Reed, A. R. Sharrett, A. L. Gross

**Affiliations:** 1 Department of Epidemiology, Johns Hopkins Bloomberg School of Public Health, 615 N. Wolfe St, Baltimore, MD USA; 2 Cochlear Center for Hearing and Public Health, Johns Hopkins Bloomberg School of Public Health, Baltimore, MD USA; 3 Wilmer Eye Institute, Johns Hopkins Hospital, Baltimore, MD USA; 4 Department of Epidemiology, School of Public Health, University of Colorado Denver, Denver, CO USA; 5 Department of Psychiatry and Human Behavior, Brown University Warren Alpert Medical School, Providence, RI USA; 6 Department of Biostatistics, Johns Hopkins Bloomberg School of Public Health, Baltimore, MD USA; 7 Department of Mental Health, Johns Hopkins Bloomberg School of Public Health, Baltimore, MD USA; 8 Memory Impairment and Neurodegenerative Dementia Center, University of Mississippi Medical Center, Jackson, MS USA

**Keywords:** Cognition, Item response theory, Measurement, Differential item functioning

## Abstract

**Background:**

Item response theory (IRT) methods for addressing differential item functioning (DIF) can detect group differences in responses to individual items (e.g., bias). IRT and DIF-detection methods have been used increasingly often to identify bias in cognitive test performance by characteristics (DIF grouping variables) such as hearing impairment, race, and educational attainment. Previous analyses have not considered the effect of missing data on inferences, although levels of missing cognitive data can be substantial in epidemiologic studies.

**Methods:**

We used data from Visit 6 (2016–2017) of the Atherosclerosis Risk in Communities Neurocognitive Study (*N* = 3,580) to explicate the effect of artificially imposed missing data patterns and imputation on DIF detection.

**Results:**

When missing data was imposed among individuals in a specific DIF group but was unrelated to cognitive test performance, there was no systematic error. However, when missing data was related to cognitive test performance and DIF group membership, there was systematic error in DIF detection. Given this missing data pattern, the median DIF detection error associated with 10%, 30%, and 50% missingness was -0.03, -0.08, and -0.14 standard deviation (SD) units without imputation, but this decreased to -0.02, -0.04, and -0.08 SD units with multiple imputation.

**Conclusions:**

Incorrect inferences in DIF testing have downstream consequences for the use of cognitive tests in research. It is therefore crucial to consider the effect and reasons behind missing data when evaluating bias in cognitive testing.

**Supplementary Information:**

The online version contains supplementary material available at 10.1186/s12874-022-01572-2.

## Background

Cognitive test scores from neuropsychological batteries are ubiquitous in studies of cognitive aging and dementia. Although neuropsychological batteries are considered the gold standard measure of cognition, careful analysis is required in light of issues such as measurement error and missing data. As an indirect reflection of underlying cognitive functioning, cognitive test scores are highly susceptible to measurement error [1, 2]. As compared to other commonly used methods for the analysis of cognitive test data (i.e., individual test scores or the generation of summary scores by averaging individual test z-scores), Item Response Theory (IRT)-based approaches to the analysis of cognitive test data offer unique advantages in quantifying and accounting for potential group differences in test responses among persons of equivalent underlying status, which can be interpreted as biases [3, 4].

In particular, IRT-based methods for the detection of differential item functioning (DIF) allow for the quantification of and adjustment for bias in cognitive tests [5–8]. In this framework, bias is indicated when there is evidence of DIF, i.e. an item on a cognitive test is easier or harder for a particular subgroup of individuals (those with hearing impairment, for example), after controlling for underlying cognitive ability [9, 10]. This situation is akin to differential measurement error in that factors hypothesized to impact underlying cognition may also differentially affect the measurement of cognition [11].

For instance, hearing impairment is hypothesized to be causally related to poor cognition through mechanisms such as cognitive load or increased social isolation [12, 13]. Simultaneously, hearing impairment may lead to bias in cognitive testing if the completion of cognitive test items depends on one’s hearing ability independently of their cognitive capacity [14]. Similarly, factors such as educational attainment and race may lead to bias in cognitive testing through increased familiarity with test-taking procedures or cultural components of tests [15, 16]. When seeking to assess the association between true underlying cognitive functioning and hearing impairment status, race, or education, a failure to account for bias in cognitive testing could lead to biased estimates. However, if DIF methods are used to identify bias in cognitive testing, investigators can choose to either remove problematic test items or adjust the measurement of cognition for the identified bias using IRT-based methods, which either use a concurrent scaling approach based on partial measurement invariance or leverage linking methods robust to the presence of DIF [17].

In addition to the highlighted challenges related to measurement error, missing data on cognitive tests can be somewhat common due to administrative procedures or participant non-response associated with task difficulty, education level, or other cultural factors [18]. While missing data can affect findings from traditional prevalence studies or risk factor analyses, to our knowledge no prior work has delineated the impact of missing data on IRT-based approaches to DIF detection and the identification of bias in cognitive test items [19, 20]. Additionally, while various imputation techniques for cognitive data exist and have been the focus of prior work, the performance of commonly used imputation methods for use in DIF detection under plausible missingness scenarios has not been previously studied [21, 22]. Error in the detection of DIF due to missing data could lead to incorrect decisions regarding which cognitive items to exclude from a neuropsychological battery or incorrect IRT-based model specifications, ultimately affecting the estimation of cognitive ability. Therefore, it is important to understand how missing data and the imputation of missing data might affect the detection of bias using IRT-based DIF methods.

Previous work in the education or psychometrics fields has explored the impact of missing data on DIF detection using DIF detection methods such as the Mantel–Haenszel or regression-based approaches [23–25]. However, no study we are aware of focused on DIF detection in cognitive testing in epidemiologic studies older adults. Differences in the sample sizes of studies (epidemiologic studies are often smaller than educational testing datasets), the number of items available (batteries are often smaller in studies of older adults), and patterns of missingness (missingness mechanisms in older adults are often closely related to disease status, mortality, and general cognitive status) might lead to different conclusions. Additionally, prior work has not assessed the impact of missing data on the Multiple Indicators, Multiple Causes model for DIF detection, which is commonly used in the study of cognitive testing among older adults [6, 9].

This study aims to (1) describe the effect of different missing data patterns on the detection of DIF in cognitive testing with IRT methods and (2) to evaluate the effect of commonly used imputation techniques in reducing the observed bias in the estimation of DIF (DIF estimation error). For illustration, we consider DIF attributable to hearing impairment status, educational attainment, and race.

## Methods

### Description of data

We used data from the Atherosclerosis Risk in Communities Neurocognitive Study (ARIC-NCS) (*N* = 3580), a prospective cohort study which recruited individuals aged 45 to 64 years old between 1987 and 1989 [26]. Recruitment for the ARIC-NCS sample was based at four university study sites (Forsyth County, North Carolina; Jackson, Mississippi; the northwest suburbs of Minneapolis, Minnesota; and Washington County, Maryland). Our primary analyses used cross-sectional data from Visit 6 (2016–2017) of ARIC-NCS. We limited the sample to participants with data for at least one cognitive test item. For the assessment of DIF by race we excluded individuals who were not of white or black race (*n* = 11).

All participants in the studies provided written informed consent. Study protocols were approved by the Institutional Review Boards at Johns Hopkins University, Wake Forest Baptist Medical Center, University of Mississippi Medical Center, and the University of Mississippi Medical Center. All data analysis projects, including this study, fall under the ARIC Data Repository Project, which has been approved by the Johns Hopkins University School of Public Health Institutional Review Board. All analyses were performed in accordance with relevant guidelines and regulations.

### Measurement of cognition

ARIC-NCS included a comprehensive neuropsychological battery testing various cognitive domains, e.g., language, attention, executive functioning, and memory. Language was assessed using the Boston Naming Test and semantic and phonemic fluency [27, 28]. The Trail-Making test part A assessed attentional ability [29]. The Trail-Making test part B, along with the WAIS-R digits backwards task, and the digit symbol substitution task were used to capture executive functioning [30–32]. Finally, memory was assessed using a delayed recall test and the Wechsler Memory Scale Revised paragraph recall test, as well as a test of incidental learning from the previously administered digit symbol substitution task [33, 34].

### Measurement of factors potentially causing differential measurement

We chose to evaluate DIF by three example grouping variables known to be associated with lower scores on cognitive tests and for which there exist plausible mechanisms for bias on cognitive testing: hearing impairment status, educational attainment, and race [35–37]. We used pure-tone audiometry to measure hearing. We defined hearing impairment as having a 4-frequency (0.5, 1, 2, 4 kHz) pure-tone average in the better hearing ear of 25 decibels hearing level or higher to create a dichotomous classification [38]. Moderate to low educational attainment was defined as high school (or equivalent) or less. For the assessment of DIF by race, we compared participants of black race, most of whom were from the Jackson, MS site, to the reference category of white race (other racial categories were excluded).

### Missingness scenarios

To examine the effect of missing data on the detection of DIF, we started with complete data and imposed two sets of scenarios with missing data. We then compared these scenarios to the reference scenario with no missing data. In both sets of scenarios, we imposed missing data only among participants in the subgroup of the DIF grouping variables more likely to have lower cognitive test scores based on prior literature (individuals with hearing impairment, with moderate to low educational attainment, and of black race) [36, 39, 40]. In our first set of scenarios (scenario set 1), missingness in a cognitive test item was randomly imposed among those in the subgroup more likely to have lower cognitive test scores (Missing at Random [MAR]). In this scenario missingness is related only to the DIF grouping variable. In the second set of scenarios (scenario set 2), missingness in a cognitive test item was imposed randomly among those in the subgroup more likely to have lower cognitive test scores and who scored below the median score on that cognitive test item (Missing Not at Random [MNAR]). To summarize, missingness is related only to the DIF grouping variable in the first set of scenarios, but in the second set of scenarios, missingness is related to both the DIF grouping variable and cognitive test score. For each scenario set, we imposed levels of 10%, 30%, and 50% missingness.

Every cognitive test item in ARIC-NCS had some nominal level of missingness (1% to 10%) (Table 1). We followed common practice whereby DIF detection is conducted on each cognitive test item independently: therefore, to maximize the available sample size for the detection of DIF, we created separate reference datasets for each cognitive test item. The dataset for the evaluation of DIF in a given cognitive test item excluded records with missing data for the item in question, although missingness on other items was allowed. For this reason, the actual sample size for detection of DIF varied slightly from item to item. Table 1 shows the percentage of missing data by item and the number of records with missing data, which were excluded in models to assess DIF on that specific cognitive test item.

**Table 1:** Demographic characteristics and the percentage of missing data on cognitive test scores in the Atherosclerosis Risk in Communities Neurocognitive Study (ARIC-NCS), 2016–2017

	**ARIC-NCS**
Age—Mean (SD)	79.8 (4.7)
Sex—Female—N(%)	2142 (59.8)
Race—Black—N (%)	837 (23.4)
Hearing Impairment—Impaired—N (%)	2614 (73.0)
Vision Impairment—Impaired—N (%)	180 (17.9)
Education: Less than HS—N (%)	428 (12.0)
Education: HS or equivalent—N (%)	1475 (41.2)
Education: Beyond HS—N (%)	1673 (46.8)
Cognitive Items—% Missing in Observed Data (N)	
Boston Naming Test (30 item)	8.2% (270)
Category Fluency (Animals)	0.5% (17)
Delayed Word Recall	1.6% (58)
Digit Symbol Backwards	8.8% (289)
Digit Symbol Substitution Task	3.9% (136)
Incidental Learning	4.5% (154)
Logical Memory 1	9.2% (303)
Logical Memory 2	9.3% (306)
Phonemic Fluency (Sum of 3 Trials)	1.5% (54)
Trail-Making Test A	3.8% (130)
Trail-Making Test B	19.1% (573)

*HS* High school, the sample size of each DIF analysis can be calculated by subtracting the number of records with missing data on a given cognitive test from the total sample size, as each DIF analysis started from the reference scenario of no missing data.

### Imputation data and methods

We evaluated commonly used imputation methods for minimizing the error due to missing data. To impute missing cognitive test scores, we used demographic variables (age, sex, race [black/white]), educational attainment, hearing and vision impairment status, all other cognitive test scores, and cognitive test scores from the prior study visit. Vision impairment was defined as presenting better-eye visual acuity worse than 20/40 [41]. Data on prior cognitive test scores was available for 3,425/3,580 (96%) of participants. We used two different imputation techniques. First, we implemented a k-nearest neighbors hot deck imputation approach, a single imputation technique that randomly samples a value from the *k*-closest records based on Gower’s similarity coefficient [42, 43]. For this analysis, we used *k* = 5, meaning the final donor record was selected from a pool of the five most similar records. Second, we used Multiple Imputation by Chained Equations (MICE) as a multiple imputation technique to impute 10 datasets [44]. Continuous variables were imputed using predictive mean matching, binary variables were imputed using logistic regression models, and ordered categorical variables were imputed using a proportional odds model.

### DIF testing

**Fig. 1:**
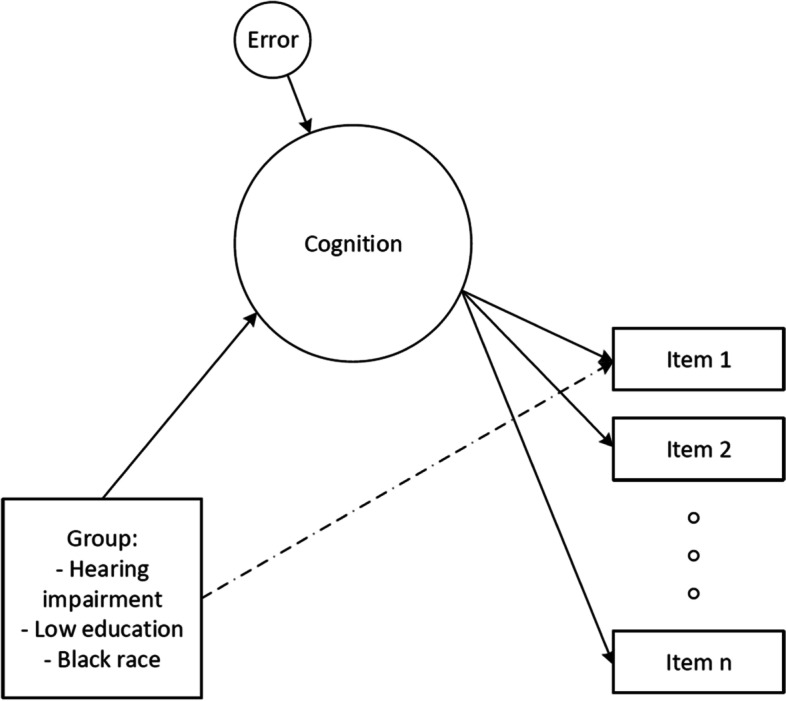
Schematic showing the relationships modeled in the Multiple Indicators, Multiple Causes (MIMIC) model. Underlying cognition leads to cognitive test performance on each of the items included in the cognitive battery. Group can influence underlying cognition, which is also influenced by some error. The dashed arrow connecting group directly to Item 1 is the primary association of interest and represents bias, or the relationship between group and cognitive test item performance, holding underlying cognition constant

To evaluate DIF we used the Multiple Indicators, Multiple Causes (MIMIC) confirmatory factor analysis model (Fig. 1) [25, 45]. While the MIMIC model is a confirmatory factor analysis model, the model functions similarly to an IRT model, which models the relationships between scores of each of the cognitive tests and underlying cognitive functioning. The MIMIC model additionally describes both (1) the association of a grouping variable on underlying latent cognition and (2) the association of a grouping variable on the score of a specific cognitive item (Fig. 1), after accounting for latent cognition. A large, significant effect (*p* < 0.05) for relationship 2 (between a grouping variable and a specific cognitive item) is an indication that the item has a relationship with the grouping variable, after controlling for underlying level of cognition (as informed by all other test items in the model) that is over and above that expected by the effect of the grouping variable on the construct of interest (relationship 1). This situation is consistent with the notion of bias in that specific test item. Bias can therefore be defined as the existence of a relationship between the grouping variable and a specific test item, controlling for underlying cognitive functioning.

A negative DIF estimate (the association between a grouping variance and an indicator) indicates that a given test item is easier for the group of interest (i.e. those with hearing impairment), whereas a positive DIF estimate indicates that a given test item is more difficult for the subgroup of interest. Because the MIMIC model used a probit link, the DIF estimate is in the unit of probits. In this analysis, we scaled models to have a mean latent cognitive score of 0 and a variance of 1, and we therefore we refer to DIF estimates and the DIF estimation error (bias in the estimation of DIF) in terms of standard deviation (SD) units. For ordinal categorical variables, MIMIC models evaluate the association between the grouping variable and performance of at least the level of each item threshold, where an item threshold represents the difficulty of scoring one category higher on a given ordinal test item. Each cognitive test item with *k* categorical response options will have *k*-1 different thresholds.

### Statistical analysis

We first summarized the ARIC-NCS sample using descriptive statistics. For each of the DIF grouping variables and cognitive test items analyzed, we performed the following 6 steps: (1) we discretized each cognitive test item using equal interval discretization into up to 8 different categories and collapsed adjoining categories if one category had fewer than 5% of the records, or if any test item had fewer than 5 records in each of the two categories defined by the DIF grouping variable, (2) we evaluated DIF at each threshold of an item given the reference scenario of no missing data, (3) we imposed levels of 10%, 30%, and 50% missingness according to both missing data scenario sets described earlier, (4) we evaluated DIF in the presence of each missing data scenario, (5) we conducted both hot deck single imputation and MICE to account for the missing data, and (6) we evaluated DIF in the presence of imputations. We then compared the difference between reference DIF estimates and DIF estimates with missing data and/or imputed data across all cognitive test items and item thresholds considered using two metrics: median absolute difference and proportion of flipped inferences. The median absolute difference was calculated as the absolute value of the difference in DIF estimates between the reference scenario and the different missingness scenarios. We chose to use the median difference so that the overall measure would not be affected by a small number of cases where large shifts could be attributed to small cell sizes. A positive difference indicates that the DIF estimate in the missingness scenario was larger than the DIF estimate in the reference scenario and a negative difference indicates that the DIF estimate in the missingness scenario was smaller than the DIF estimate in the reference scenario. The proportion of DIF estimates with flipped inferences was defined as the proportion of estimates for which the statistical significance (at the α = 0.05 level) of the reference scenario DIF estimate was discrepant with the statistical significance of the DIF estimate in the scenario with missing or imputed data, or an instance when the two estimates were both statistically significant but in opposite directions.

In a post-hoc exploratory analysis to explore potential reasons behind differences in the performance of hot deck single imputation and MICE, we compared the median absolute difference in DIF estimates between the reference scenario and scenarios with missing data imputed with either hot deck single imputation or MICE to an additional scenario where missing data were imputed using single imputation by chained equations.

Statistical analyses were conducted in STATA 15 or R version 4.0.2. Hot deck single imputations were conducted with the simputation R package [46] and MICE was conducted with the mice R package [47]. All MIMIC models were conducted using a maximum likelihood (MLR) estimator in Mplus version 8 and the MplusAutomation R package [48].

## Results

### Study characteristics

In ARIC-NCS, participants had a mean age of 79.8 years (standard deviation [SD] = 4.7), and 59.8% of the sample was female (Table 1). We examined the effect of missing data on three DIF grouping variables: hearing impairment (73.0% of the sample), moderate to low educational attainment (53.2%), and black race (23.4%). The proportion of missing data on cognitive tests was mostly 1% to 10% but ranged up to 19.1% (Trail-making test B).

### Effect of missing data on DIF estimates

**Fig. 2:**
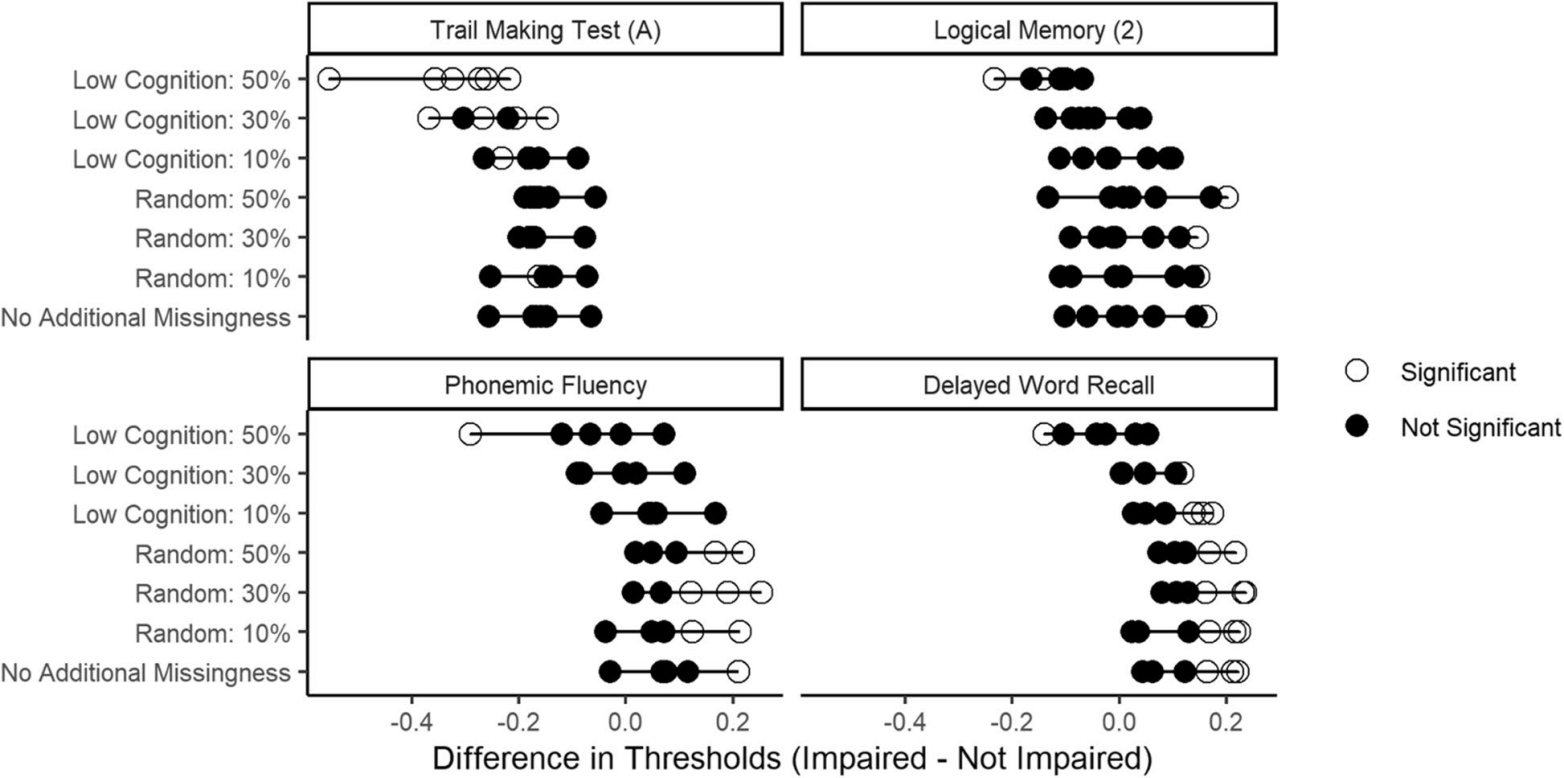
DIF estimates for hearing impairment in the reference scenario (No Additional Missingness) compared to the six missingness scenarios for select cognitive test items in the Atherosclerosis Risk in Communities Neurocognitive Study (ARIC-NCS). Each dot represents a difference between the two groups (impaired and unimpaired) for a single threshold of the ordinal test score. When the difference in thresholds is significantly different from zero (indicated by an unfilled circle), this indicates DIF. Differences between the reference scenario and each of the missingness scenarios illustrates the magnitude of error due to missingness. Estimated thresholds in the random scenarios are generally stacked vertically on top of the estimates with no additional missingness, indicating no systematic DIF estimation error. However, estimates for scenarios with missingness among lower cognitive test scores are shifted to the left, indicating systematic DIF estimation error in these scenarios

When examining the effect of missing data on the detection of DIF by hearing impairment, we found that when data were missing at random with respect to cognitive test score (scenario set 1), DIF estimation error was small and random. However, when missing data was imposed only among those with low cognitive test scores (scenario set 2), there was systematic DIF estimation error. For example, in the phonemic fluency task, the median DIF estimation error due to random missingness across the thresholds of the item was 0.00, 0.04, and 0.02 SD units in the 10%, 30%, and 50% missing data scenarios, suggesting no systematic error. However, the respective DIF estimation error due to missingness in low cognitive test scores was -0.03, -0.08, and -0.14 SD units, indicating worsening by approximately 0.03 units per 10% additional missing data. These patterns can be seen visually for select items in Fig. 2, which shows the DIF estimates for the reference scenario of no missing data in comparison to the DIF estimates in each missing data scenario. For the random missing data scenarios (scenario set 1), the DIF estimates align with the reference scenario closely on the x-axis, indicating no systematic error, whereas for the scenarios with missing data related to low cognitive performance (scenario set 2) the DIF estimates all show negative error, with larger discrepancies from the reference estimate in the scenarios with a greater amount of missing data.

**Fig. 3:**
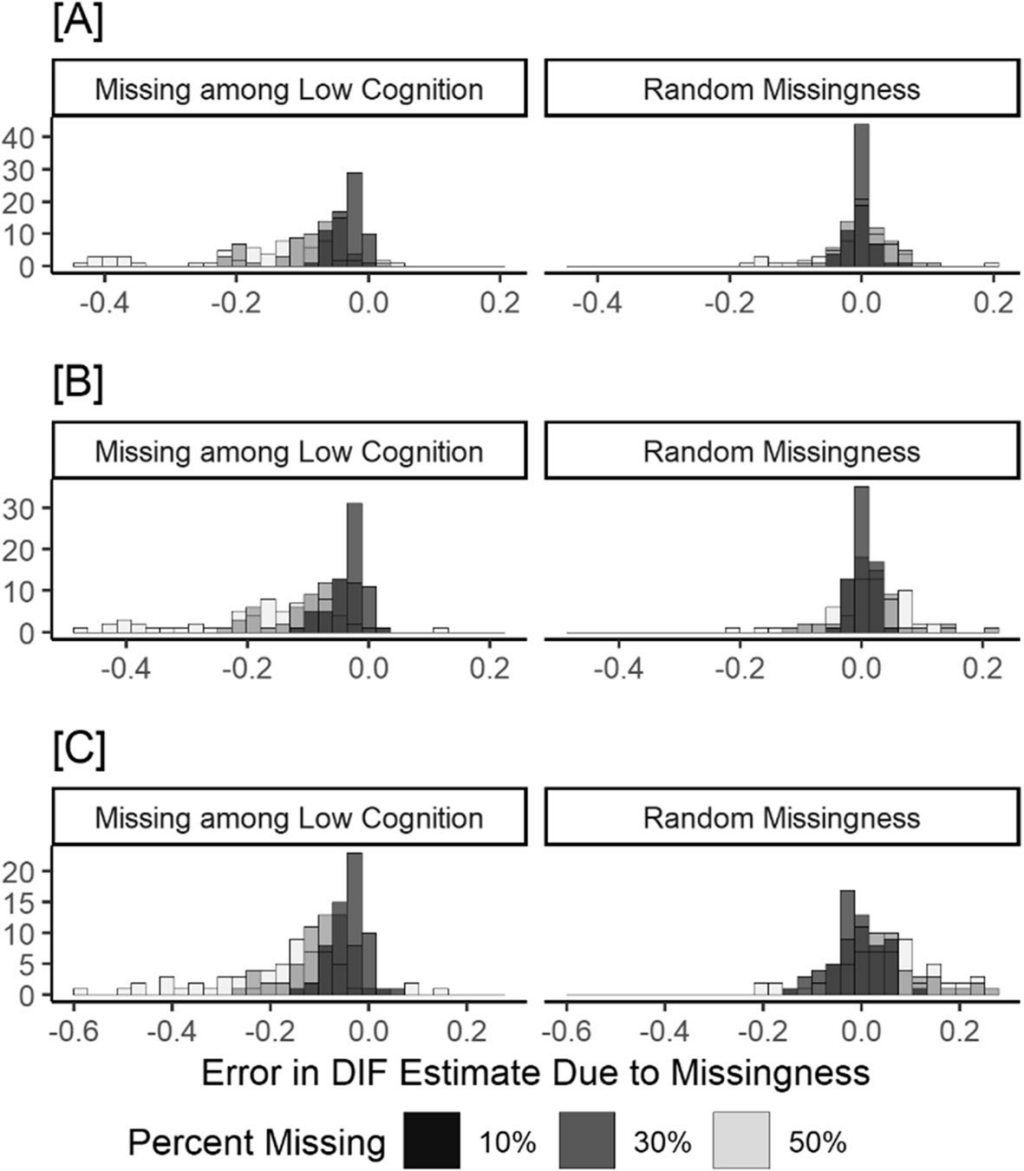
Distributions of errors in DIF estimates for (**A**) hearing impairment, (**B**) moderate to low education, and (**C**) black race due to different missing data scenarios across all cognitive tests and thresholds in the Atherosclerosis Risk in Communities Neurocognitive Study (ARIC-NCS)

The patterns of errors attributable to both sets of missing data scenarios were similar across the three different DIF grouping variables when examining the distributions of estimates across all cognitive test items (Fig. 3). In our analysis, if the original DIF estimate was negative (suggesting that the test item was easier in the impaired group), then the error due to missingness would lead to a more extreme estimate in the same direction. However, if the original DIF estimate was positive (suggesting that the test item was harder in the impaired group), then the error due to missingness could lead to a less extreme estimate in the same direction, a null estimate, or an estimate in the opposite direction.

### Effect of imputing missing data

**Fig. 4:**
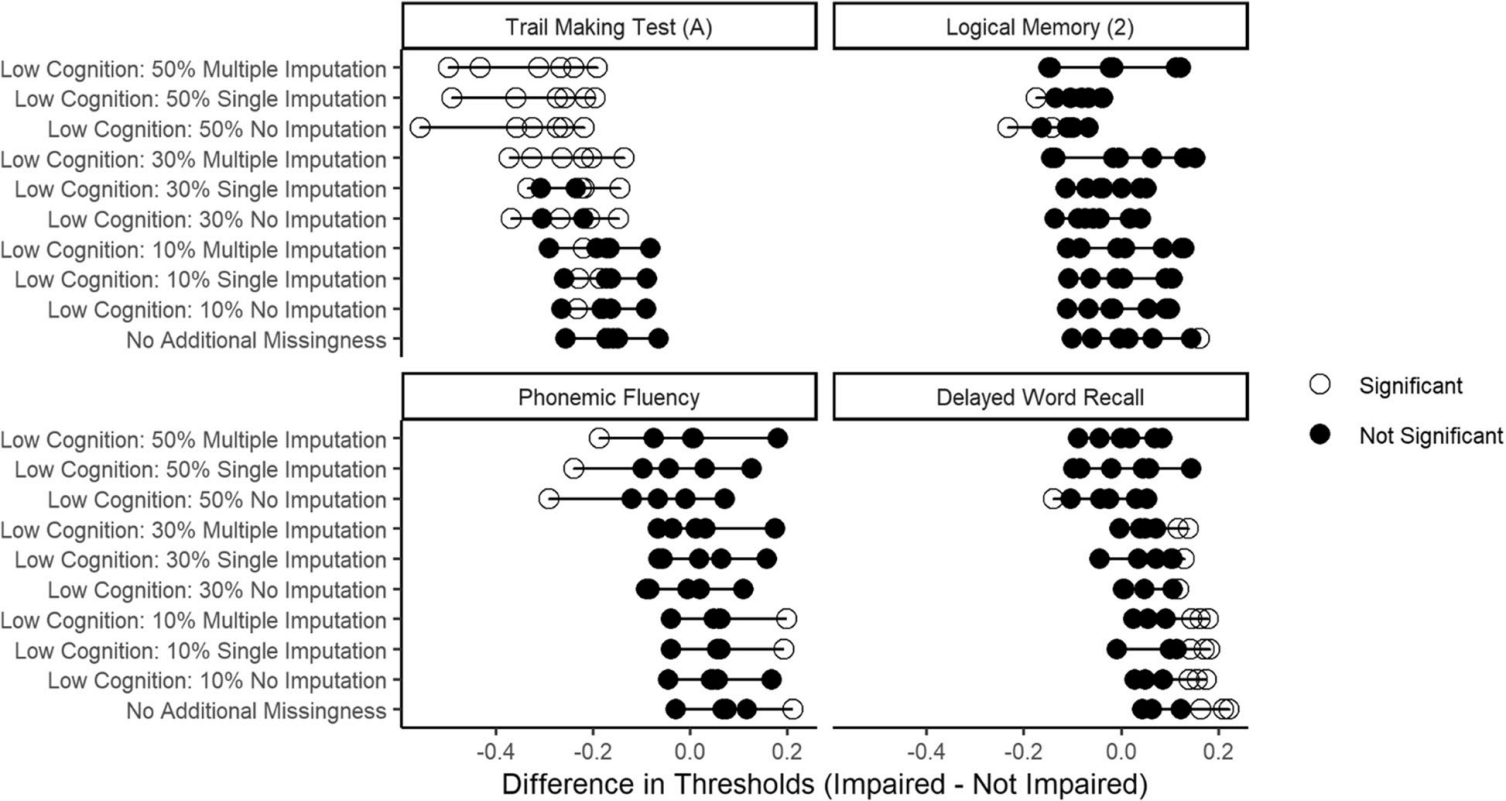
DIF estimates for hearing impairment in the reference scenario (No Additional Missingness) compared to low missingness scenarios with and without hot deck single imputation (single imputation) and Multiple Imputation by Chained Equations (MICE) (multiple imputation) for select cognitive test items in the Atherosclerosis Risk in Communities Neurocognitive Study (ARIC-NCS). Each dot represents a difference between the two groups (impaired and unimpaired) for a single threshold of the ordinal test score. When the difference in thresholds is significantly different from zero (indicated by an unfilled circle), this indicates DIF. Differences between the reference scenario and each of the missingness scenarios illustrates the magnitude of error due to missingness. The scenarios with no imputation are shifted to the left as compared to the reference estimates with no additional missingness, indicating systematic DIF estimation error. However, estimates with both single and multiple imputation more closely align with the reference estimates, illustrating the reduction of DIF estimation error with both imputation strategies

Because we only observed evidence of systematic DIF estimation error when missingness was among low cognitive scores (scenario set 2), we limited our analysis on the effect of imputation to these missingness scenarios. Both hot deck single imputation and MICE helped recover the reference DIF estimates and reduced the magnitude of the DIF estimation error in the scenarios with no imputation. Returning to the example of DIF by hearing impairment in the phonemic fluency task, where the DIF estimation error due to missingness among low cognitive scores (scenario set 2) without imputation was -0.03, -0.08 and -0.14 SD units with 10%, 30%, and 50% missingness, these errors were reduced to -0.02, -0.05, and -0.09 SD units with hot deck single imputation and -0.02, -0.05, and -0.06 SD units with MICE (Fig. 4).

**Fig. 5:**
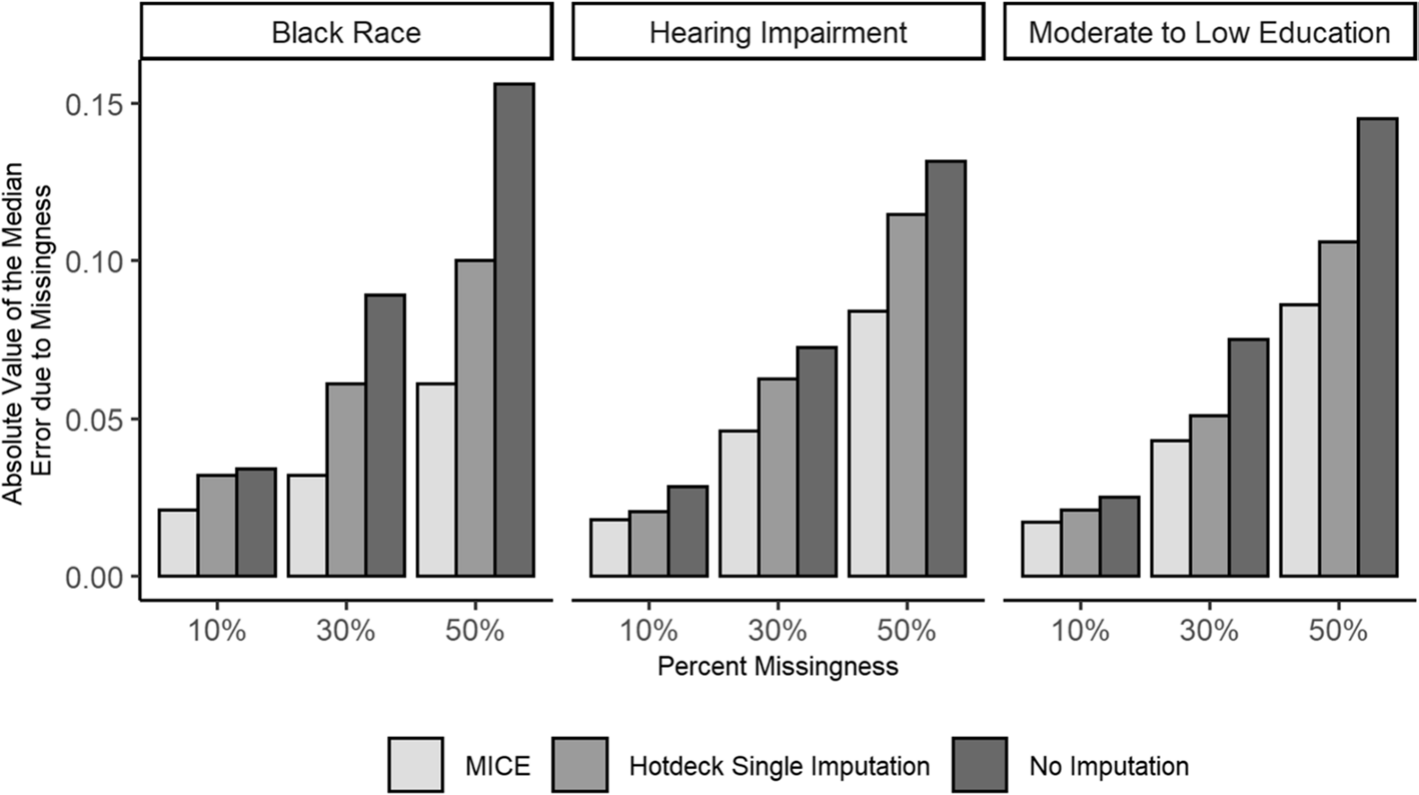
Absolute value of the median error in DIF estimates due to missingness related to cognitive test performance for hearing impairment, black race and moderate to low education in the Atherosclerosis Risk in Communities Neurocognitive Study (ARIC-NCS). Estimates are shown for scenarios with no imputation, hot deck single imputation, and Multiple Imputation by Chained Equations (MICE)

When examining the effect of imputation on the magnitude of DIF estimation error due to missingness among those with low cognition across all cognitive test items, the patterns observed across the three DIF grouping variables considered were fairly consistent (Fig. 5). In almost all instances, both imputation methods lowered the magnitude of the median error observed, but MICE consistently outperformed hot deck single imputation in minimizing DIF estimation error. Summarizing across all of the test items and DIF grouping variables considered, the median error due to 10%, 30%, and 50% missingness was -0.03, -0.08, and -0.14 SD units with no imputation, as opposed to -0.02, -0.06, and -0.11 SD units with hot deck single imputation, and -0.02, -0.04, and -0.08 SD units with MICE. The median error due to missingness for single imputation by chained equations was lower compared to hot deck single imputation and similar to MICE (Additional File 1).

**Fig. 6:**
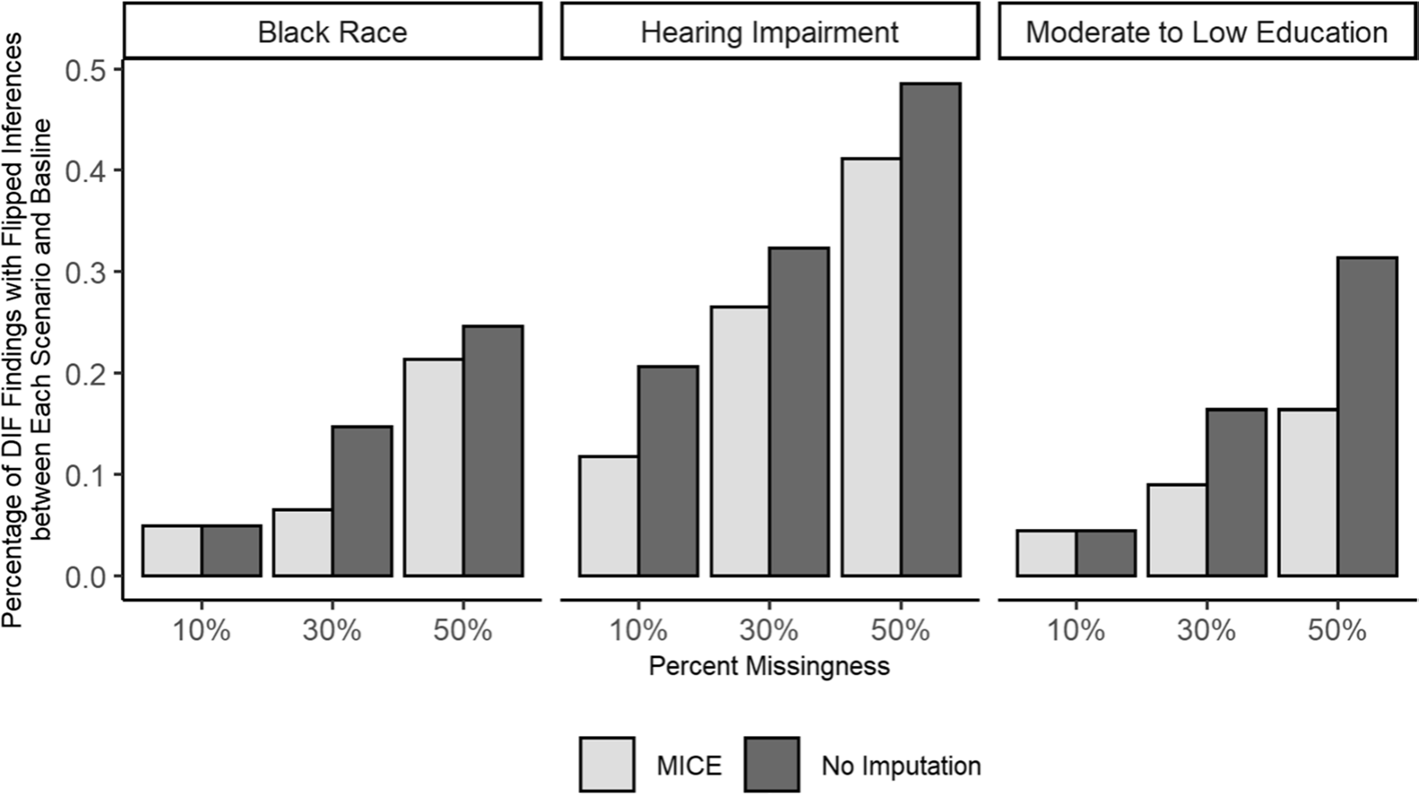
Percentage of DIF estimates with flipped inferences due to missingness related to cognitive test performance for hearing impairment, black race and moderate to low education in the Atherosclerosis Risk in Communities Neurocognitive Study (ARIC-NCS). Estimates are shown for scenarios with no imputation and Multiple Imputation by Chained Equations (MICE). Inferences are considered flipped if the statistical significance of the estimate at the *α* = 0.05 level is discrepant with the statistical significance of the reference estimate without missing data

In many cases, the DIF estimation error due to missing data among those with low cognition led to changes in inferences regarding the significance of DIF estimates at the α = 0.05 level. However, implementing MICE resulted in a reduction of the proportion of inferences that were flipped compared to reference DIF estimates (Fig. 6). As single imputation does not produce valid standard errors, hot deck single imputation had minimal effects. Overall, across all of the cognitive test items and DIF grouping variables considered, the proportion of DIF estimates with flipped inferences due to 10%, 30%, and 50% missing data related to cognitive test performance was 10%, 21%, and 35% with no imputation, 10%, 18%, and 33% with hot deck single imputation, and 7%, 14%, and 27% with MICE.

## Discussion

We found that missingness related to the DIF grouping variable but not to cognitive test scores led to random DIF estimation error, but that missingness related to both the DIF grouping variable and cognitive test scores led to systematic DIF estimation error. Our results suggest the importance of evaluating missing data in cognitive test items when assessing bias in cognitive testing using DIF methods, especially when missingness is differential by level of cognitive functioning. In particular, when the proportion of data missing is larger than 10%, we observed much larger magnitudes of DIF estimation error.

MICE performed slightly better than hot deck single imputation in reducing observed DIF estimation error, although both methods did reduce observed error compared to scenarios with no imputation. Differences could be attributed to differences in the method of imputation rather than number of imputations. Only MICE prevented flipped or incorrect inferences. When the proportion of data missing exceeds the 10% threshold, we recommend MICE as a method to reduce the bias due to missing data when testing for DIF. However, in our analyses, MICE was unable to fully eliminate the observed error due to missing data. This stands to reason considering that standard imputation methods are not equipped to correct missingness not at random. Therefore, we suggest that investigators interpret results cautiously even when using MICE to address missing cognitive data.

Assuming that individuals in the group with lower average cognitive test performances (e.g. those with hearing impairment, moderate to low educational attainment, and black race), were also more likely to have missing data, the resulting error was in the negative direction. Therefore, depending on the true DIF estimate, the error could be towards the null, away from the null, or could even lead to incorrect inferences. For example, if a given cognitive test item has a no bias and a true DIF finding of 0, in the presence of missing data, the DIF estimate may be negative, indicating the item is easier for the impaired group. Alternatively, if a given cognitive test item has bias and the true DIF finding is positive (the item is harder for the impaired group), in the presence of missing data the DIF estimate may be null or even negative in extreme cases (no bias or the item is easier for the impaired group). The observed pattern of results was consistent across all cognitive test items considered, and where identified, the magnitude of systematic bias showed a dose–response relationship with the magnitude of missing data. This consistency increases confidence in findings despite the smaller sample size of our real-world dataset.

Previous work has shown that scores from an IRT-based approach and scores from the commonly used approach of taking the average of cognitive test z-scores are highly correlated, but that despite this high correlation the IRT approach is less biased, has more power to detect changes in cognition over time, and has higher criterion validity [49]. Additionally, IRT methods allow for DIF detection and adjustment for DIF, which are otherwise not possible in an empirical, principled manner. When DIF is present, DIF adjustment can sometimes lead to large changes in the estimated cognitive ability of individuals, but even small changes can be important when an individual’s score lies near a cut-point used to define impairment [50]. The evaluation and adjustment for bias is most important and will have the greatest impact on the overall study conclusions for analyses that seek to evaluate the effect of risk factors that also lead to testing bias or are also associated with factors that lead to biases in cognitive testing [11].

In settings where potential risk factors also are associated with biases in cognitive testing, it is of particular importance that IRT-based methods are leveraged to evaluate bias, and that the extent of missing data and the potential effect of missing data is considered in conducting DIF analyses. When the proportion of missing data exceeds 10% and it is plausible that missing data may be related to cognitive test scores, MICE should be considered to reduce potential error due to missing data and prevent incorrect decisions regarding DIF and DIF adjustment. Recognizing that imputation cannot be fully effective when missing data mechanisms are informative, extreme caution should be exercised in deciding to include items in analyses whose missing data percentage is considerable. Auxiliary information not of interest for the primary analysis, but that may relate strongly to missing data mechanisms, should be incorporated where possible. Incorrect decisions regarding the presence of DIF and the need for DIF adjustment can lead to differences in estimated cognitive scores and differences in the results and inferences of epidemiologic studies. It is worthwhile to note, however, that we evaluated levels of missing data from 10 to 50%. These magnitudes of missing data are somewhat rare in well-conducted epidemiologic studies: in ARIC-NCS, missingness in any test item, with one exception, was mostly 1% to 10%. Where the magnitude of missing data in studies is lower than 10%, the magnitude of systematic DIF estimation error and the impact on inferences in DIF detection due to missing data will likely be small.

Some limitations should be considered. First, we evaluated the effect of missingness on DIF detection using one strategy (MIMIC models) for assessing DIF, but other methods exist [51, 52]. While DIF detection methods are fairly similar, they test slightly different hypotheses and can lead to different conclusions at times [53, 54]. Therefore, our results may not be generalizable to other methods of assessing DIF. Additionally, we implemented the most common DIF methodology of assessing one item at a time, however, the effect of missing data on less biased synthetic approaches to evaluating DIF in multiple items concurrently should be explored [55]. Second, while we evaluated three DIF grouping variables to illustrate multiple examples, we considered these grouping variables separately, whereas a substantive analysis focused on multiple, correlated DIF grouping variables would likely prefer to evaluate conditional DIF. Moreover, we limited this analysis to dichotomous DIF variables; other methods allow for more flexible models and can estimate DIF effects for continuous variables, or for multiple variables simultaneously [56].

Third, we used the same set of imputations across analyses, relying on a limited set of variables for imputing cognitive data, including other cognitive test scores, prior cognitive test performance and basic demographic variables. It may be possible to achieve better performance (greater reduction of the DIF estimation error) through the addition of other health-related variables or interaction terms. In particular, given a specific exposure of interest, one should either use interaction-terms or separate imputation models by that exposure variable to ensure that imputations accurately capture differences in missingness patterns between the exposure groups. Fourth, we focused on commonly used imputation methods, but these methods are not designed to be used when data are missing not at random. Methods for the imputation of data missing not at random exist and the implementation of these methods may serve to further reduce the observed error due to missing data [57, 58]. Future work should focus on understanding how the use of these methods affects DIF estimation error due to missing data.

## Conclusions

IRT methods allow for the quantification of and adjustment for bias in cognitive testing through testing for and adjusting for DIF, which can be crucial when an analysis seeks to evaluate a risk factor that may be associated with bias in test scores. However, this analysis illustrated how missing data can affect the results of DIF detection; care should be taken to evaluate missing data in cognitive test scores before DIF analyses. Further, multiple imputation methods should be considered to reduce the potential DIF estimation error, particularly when levels of missing data are high and related to the cognitive outcome of interest.

## Supplementary Information

**Additional file 1:** **Supplementary Figure**. Absolute value of the median error in DIF estimates due to missingnessrelated to cognitive test performance for hearing impairment, black race andmoderate to low education in the Atherosclerosis Risk in CommunitiesNeurocognitive Study (ARIC-NCS). Estimates are shown for scenarios with noimputation, hotdeck single imputation, Single Imputation by Chained Equations(SICE) and Multiple Imputation by Chained Equations (MICE).

## Data Availability

We used de-identified data from the Atherosclerosis Risk in Communities Neurocognitive Study. Researchers can apply to access these data at https://sites.cscc.unc.edu/aric/opportunities_for_new_investigators.

## Abbreviations

IRT: Item Response Theory
DIF: Differential Item Functioning
SD: Standard Deviation
ARIC-NCS: Atherosclerosis Risk in Communities Neurocognitive Study
MAR: Missing At Random
MNAR: Missing Not At Random
MICE: Multiple Imputation by Chained Equations
MIMIC: Multiple Indicators, Multiple Causes
MLR: Maximum Likelihood, Robust
