# Supplementary material for: The effect of missing data and imputation on the detection of bias in cognitive testing using differential item functioning methods
Source: BMC Med Res Methodol. 2022 Mar 27;22:81. doi: 10.1186/s12874-022-01572-2 (PMC8961895; doi:10.1186/s12874-022-01572-2)
Supplement: Supplementary file 1 — Additional file 1: Supplementary Figure. Absolute value of the median error in DIF estimates due to missingnessrelated to cognitive test performance for hearing impairment, black race andmoderate to low education in the Atherosclerosis Risk in CommunitiesNeurocognitive Study (ARIC-NCS). Estimates are shown for scenarios with noimputation, hotdeck single imputation, Single Imputation by Chained Equations(SICE) and Multiple Imputation by Chained Equations (MICE). [file 12874_2022_1572_MOESM1_ESM.docx]

**Additional File 1**

Supplementary Figure. Absolute value of the median error in DIF estimates due to missingness related to cognitive test performance for hearing impairment, black race and moderate to low education in the Atherosclerosis Risk in Communities Neurocognitive Study (ARIC-NCS). Estimates are shown for scenarios with no imputation, hotdeck single imputation, Single Imputation by Chained Equations (SICE) and Multiple Imputation by Chained Equations (MICE).

**
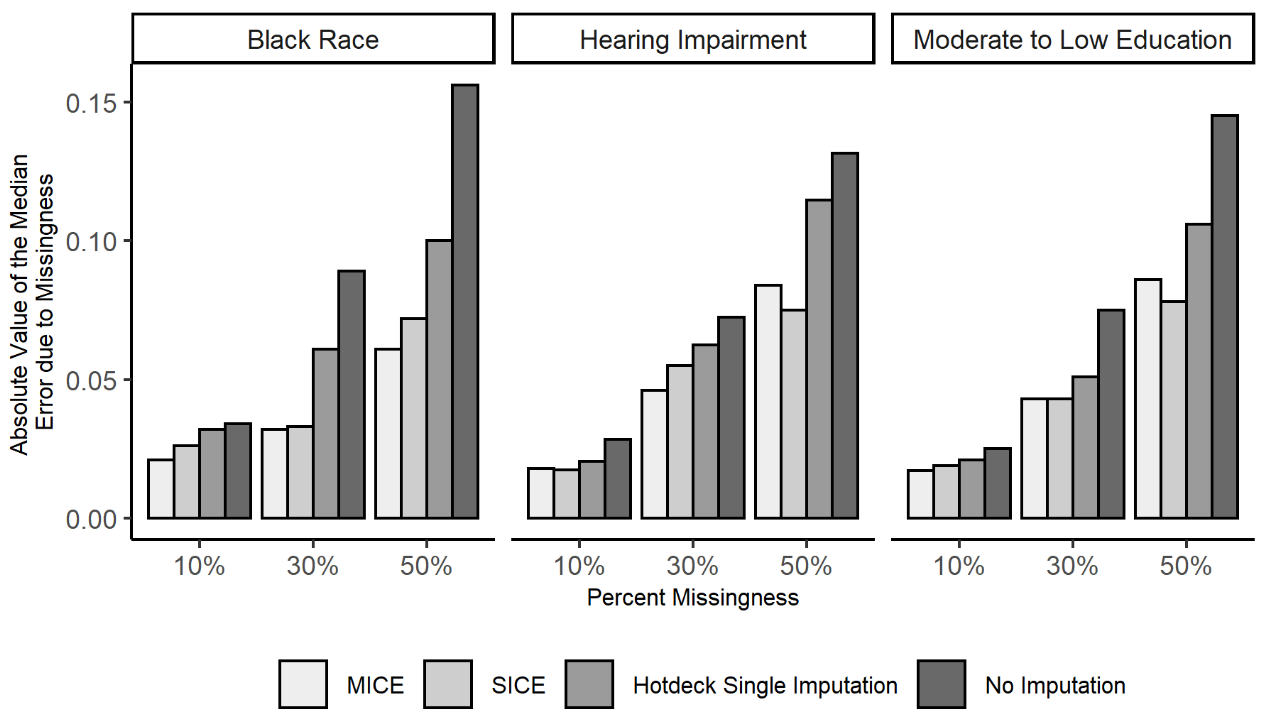
**
